# Suicide prevention gatekeeper training in the Netherlands improves gatekeepers’ knowledge of suicide prevention and their confidence to discuss suicidality, an observational study

**DOI:** 10.1186/s12889-018-5512-8

**Published:** 2018-05-18

**Authors:** Sanne Terpstra, Aartjan Beekman, Jens Abbing, Sabine Jaken, Martin Steendam, Renske Gilissen

**Affiliations:** 1 Department of Research & Innovation, GGz InGeest, Amsterdam, The Netherlands; 2 Department of Psychiatry, VU University Medical Centre, Amsterdam, The Netherlands; 3 Department of Public and Occupational Health, VU University Medical Centre, Amsterdam, The Netherlands; 4 113 Suicide Prevention, Amsterdam, The Netherlands; 5 Department of Psychology, GGz Friesland, Leeuwarden, The Netherlands

**Keywords:** Gatekeeper training, Suicide prevention, Observational study, Employment sectors

## Abstract

**Background:**

The gatekeeper training is designed to help identify suicidal individuals, respond to suicidal ideation and refer to help. The internationally widely used training shows promising results. This is the first study presenting its effectiveness in the Netherlands and the first study investigating the effect in different employment sectors.

**Methods:**

In an observational study, 113 Suicide Prevention – the Dutch suicide prevention expertise centre and lifeline - trained 526 professionals as gatekeepers. Changes in gatekeepers’ identifying and referral behaviour, knowledge of suicide prevention and skills-confidence were studied, using a pre-post (6 weeks after training) self-report questionnaire. Outcomes were analyzed with General Linear Model (GLM) repeated measures with four employment sectors (healthcare-, educational-, socioeconomic and other sectors) as a between-subjects factor.

**Results:**

Pre-post self-reports of 174 respondents showed no change in the identification of suicidal people, referrals to the general practitioner (GP) or lifeline 113, but significant improvement in professionals’ knowledge and confidence (*p* < .001). Results did not differ between employment sectors.

**Conclusions:**

The gatekeeper training significantly increases suicide prevention knowledge and skills confidence in abilities to address suicidality. Healthcare, education, socioeconomic and other professionals (e.g. security, justice, transport, church workers) benefit similarly from the training. Increasing the number of gatekeeper training programs in all sectors is recommended.

**Electronic supplementary material:**

The online version of this article (10.1186/s12889-018-5512-8) contains supplementary material, which is available to authorized users.

## Background

In the Netherlands 1893 people died by suicide in 2016 in a population of almost 17 million. In 2007 the number of suicidal deaths was 1353 [1]. After correcting for population growth, the suicide rate increased by 34% (2007:8.3 per 100,000; 2016:11.1 per 100,000). Given the increase in suicidal deaths, several actions have been undertaken in recent years to organize suicide prevention activities in mental healthcare settings and beyond. In 2012, a Multidisciplinary guideline to diagnose and treat suicidal behaviour in Dutch mental healthcare [2] was published. The number of suicides among clients of mental healthcare settings, as reported by the Dutch Ministry of Health, Welfare and Sport shows that about 50% of Dutch citizens who died by suicide were or had been in treatment [3], implying that 50% of Dutch suicides were unknown in mental healthcare settings. This shows the importance of identifying and assisting people with suicidal ideation in healthcare but also within community settings such as schools, volunteer organizations or social services.

The Dutch suicide prevention expertise organization, 113 Suicide Prevention, provides a telephonic and digital ‘lifeline’ for anonymous help. In 2013 the organization launched a National Agenda on suicide prevention with the support of the Dutch Ministry of Health. Effectuation and evaluation of the National Agenda was carried out by 113 in close collaboration with social community organizations and the national railway company (ProRail). The common goal was to contribute to a decrease in Dutch suicide rates by initiating and encouraging the realization of suicide prevention policies and other preventive activities. As there is a general consensus that the pathways to suicidality consist of a multiple factors [4, 5], the Agenda was directed at five domains: (mental) healthcare, (social) media, education, the socioeconomic and transport sector. One of the important actions undertaken was disseminating gatekeeper trainings in community settings such as schools, GP offices and social services.

Acknowledgment for the National Agenda initiative comes from the World Health Organization (WHO), recommending multiple level interventions in community environments in its report Preventing Suicide: a Global Imperative [6]. Some multi-level interventions on suicide prevention have been extensively studied and show promising results [7–15]. A desirable effect of gatekeeper trainings as part of such a program can be found in several studies [11, 16–19], showing a progressive reduction in suicide rates in the intervention communities. The effects of the training itself though, are difficult to discern since they were part of these multi-layered intervention programs. Nevertheless, besides multi-level actions, the gatekeeper training as a standalone strategy seems to bring about positive results as well [20–22], apart from resident assistants in a randomized controlled trial who showed no effects on intervention behaviour [23] after a 1-h gatekeeper training.

In most studies, knowledge improvement is a convincing outcome of the gatekeeper training [16, 24–29] as well as improved self-efficacy [25, 28, 30, 31] and confidence to act when in contact with a suicidal person [16, 24, 27, 32]. Furthermore, gatekeeper training seems beneficial in referring youth to appropriate services, especially within school-based settings [33, 34]. The improved self-efficacy found in Chauliacs controlled study resulted in significantly more referrals to psychologists [24]. On the other hand, four uncontrolled studies found that participants with higher pre-training intentions to help, were more likely to actually reach out to a person in a suicidal crisis and showed a decline in referral behaviour [26]. Heightened awareness of suicidal signs is another goal targeted by most gatekeeper trainings. Tsai’s randomized controlled trial showed that nurses’ recognition of signs significantly increased [35]. Also for clergy, after attending a gatekeeper training their knowledge, among other factors, improved and contributed to better prediction of high risk individuals [36].

Despite differences in training methodology, duration, content and target group, the general goal of gatekeeper training is to learn how to respond to a suicidal person by starting a “caring dialogue” [37] and “open the gates” [26, 38] to further help when needed, for example by referring to a mental healthcare professional [39]. Still, while gatekeeper training is a frequently used and widely assessed intervention, various studies suggest gatekeeper training programs for preventing suicide need more thorough study to determine their ultimate effectiveness [15, 16, 39, 40].

This is the first Dutch study on the effectiveness of gatekeeper training in the Netherlands. Firstly, to explore the effect of gatekeeper training content on participants in the Netherlands, but also to study to what extent sectors differ in the reception of suicide prevention training and thus gain insight into the potential of the employment sectors to suicide prevention.

### Gatekeeper training in the Netherlands

The gatekeeper training frequently referred to is based on the method of Question, Persuade, Refer (QPR) and was originally set up as a mental health intervention for those in a suicidal emergency [37]. Gatekeeper training is usually given to either designated professionals (such as counselors and GPs) specialized in helping people in crisis or to community members with facilitating jobs, not trained in dealing with a potential mental healthcare crisis (such as educational staff) [38]. These community workers can play a crucial role in responding to suicidality because they are likely to meet a diversity of people, including people who do not seek mental help themselves. In particular, employees in unemployment benefit agencies and social services have a higher probability of interacting with suicidal clients at their workplace [41].

In 2008 the gatekeeper training was tailored for application in the Netherlands [42]. Since then, a variety of professionals have been trained, such as teachers, clergymen, police-officers, social workers, general practitioners and bailiffs. In 2014 the gatekeeper training program was handed over to 113 Suicide Prevention for further dissemination.

The Dutch gatekeeper training program is designed as skills training with a duration of four hours and is based on the QPR principles [43]. It consists of four main parts: (i) introduction, (ii) theoretical background, (iii) role-plays and (iv) referral pathways. In the introduction, the topic of suicide is explored and attendees share their experiences. The theoretical background focuses on the epidemiology of suicide, risk factors and possible causes, and stresses the global consensus of experts that talking about suicidal ideation can help to prevent suicide. After this, role-plays are performed by trainees and are repeated three times in order to deepen the skills and consolidate the trainees’ confidence. Finally, there is a plenary discussion on the best routes for referral and further help.

Each training is administered by two trainers. In the time period corresponding with this study (January 2015 until July 2016) 113 Suicide Prevention worked with approximately 20 gatekeeper trainers. Almost half of them had conducted this training several times before. Trainers were employed by mental healthcare institutions, or were selected at the start of 2015 after an application procedure. They received a two-day train-the-trainer program, identical to that of the experienced trainers. Some trainers worked in mental or public healthcare, some were trainers by profession but had no experience in healthcare or with suicide prevention. The trainers trained in pairs, and pairs varied per training.

So far, in the Netherlands no studies have been conducted on the effects of gatekeeper training on suicide prevention. This study aims to fill that gap. Not only will it (1) determine the short-term effectiveness of the gatekeeper training program on individual participants in terms of a) identifying and referral behaviour; b) knowledge of suicide prevention; and c) confidence in the ability to have a dialogue about suicidality with a person with suicidal ideation. It also aims to (2) ascertain whether the training has different outcomes for different employment sectors. To our knowledge, comparison of multiple sectors in one study has not been conducted previously. Healthcare professionals might benefit less from the gatekeeper training than staff from the educational sector, the socioeconomic sector or other employment sectors, due to their prior education and thus higher pre-test abilities. As a consequence, these professionals may already be more familiar and thus more at ease discussing difficult health issues.

## Method

### Participants and procedure

One hundred thirteen Suicide Prevention contacted organizations within different employment sectors to offer the gatekeeper training. From these organizations, employees volunteered to participate in the training. The training program of 113 Suicide Prevention accepted a maximum of 16 and a minimum of 10 participants per training. From January 2015 until July 2016 113 Suicide Prevention trainers carried out 42 gatekeeper trainings (2015:23; 2016:19) and trained 526 individuals (2015: *n* = 305; 2016: *n* = 221).

On entering the training facilities, participants were asked to fill in a questionnaire (t0), prior to the start of the training. Participants who arrived on time (*N* = 502) filled in this baseline questionnaire (t0). Approximately 6 weeks after baseline, 113 Suicide Prevention sent the same questionnaire by email for follow-up (t1). When no response was received, 113 sent a digital reminder. Of all baseline responders, 174 filled in the online-survey post-training (response rate 34.7%). Responders did not differ from nonresponders on their pre-training scores: *t*_identifying_ (497) = − 1.28, *p* = .20; *t*_referralGP_ (498) = − 1.45, *p* = .15; *t*_referral113_ (208.379) = 1.22, *p* = .23; *t*_knowledge_ (470) = 0.29, *p* = .77; *t*_confidence_ (481) = − 0.65, *p* = .52.

Of all responding participants, 49 worked in the educational sector, 70 in the healthcare sector, 32 were employed in the socioeconomic sector and 23 in other sectors like security and justice, transport or churches and mosques. When comparing responders and nonresponders per sector, an equal proportion worked in the education sector (28.2% vs 24.4%), healthcare sector (40.2% vs 40.9%), socioeconomic sector (18.4% vs 22.3%) and other employment sectors (13.2% vs 12.5%).

### Measures

Prior to the training, participants were asked to fill in the date of training, name, email address and profession or professional organization. Furthermore, the questionnaires pre- and post-training consisted of a subset of ten questions (Additional file 1), derived from previously validated questionnaires. The subset was partially adopted from an original gatekeeper training survey [29], designed to study the effect of a QPR training amongst secondary school staff using a group-based randomized trial. Added to the items on knowledge were three survey questions assessing confidence on talking about suicide by healthcare staff, used in a cluster randomized controlled trial [44] and an empirical assessment and treatment study [45]. The impact of the gatekeeper training was analyzed for identifying people at risk of suicide, referral to GP, referral to 113 Suicide Prevention, knowledge and confidence.

### Analyses

Because each participant had been measured on two occasions (before and approximately six weeks after the gatekeeper training) on the same questionnaire, a GLM Repeated Measures was conducted to reveal if the training improved their identifying (question 1) and referral behaviour (question 2 and 3), their knowledge on suicide prevention (sumscore of questions 4–7) and their confidence in their abilities (sumscore of questions 8–10; research question 1). The last item in the confidence questions (I hesitate to ask someone if he/she is suicidal) was recoded so that a high score represents less hesitation to ask clients about suicidality.

To compare the training effects for participants from different sectors (research question 2), employment sector was entered as a between-subjects factor in the repeated measures analysis. Participants were categorized into four different employment sectors. The following sectors were distinguished: (1) education (student career counselors, teachers, student psychologists, school social workers); (2) healthcare (mental healthcare professionals, GPs, social workers, social community teams, first aid workers); (3) socioeconomic sector (bailiffs, vocational experts, bank employees, insurance doctors, debt counselors and administrators); (4) other (security, justice, transport, church workers, imams etc). All analyses were performed using SPSS 24.0.

## Results

Five hundred two participants filled in the questionnaire at pre-training and 174 at post-training (Response rate 35%). Response rates between different sectors varied between 30 and 38% (Table 1).

**Table 1:** Characteristics of the study population

Sector	N Pre-training	N Post-training	Response rate (%)
Education	129	49	38
Healthcare	204	70	34
Socioeconomic	105	32	30
Other	64	23	36
Total	502	174	35

Table 2 provides an overview of the pre- and post-training mean scores for each item of the questionnaire. None of the three items about identifying and referral behaviour differed significantly after training compared to before. Thus, the number of suicidal people that were spoken to by participants, the number of people that were referred to a GP or to 113 Suicide Prevention by participants, did not change after training. However, the training had highly significant positive effects (*p* < .001) on the items of knowledge and confidence, demonstrating that after being trained, participants had more knowledge about suicide and felt more equipped to talk to suicidal people and to refer them to appropriate sources for help. The standardized effect sizes (Cohen’s d) were − 0.03 (95% CI -0.32 to 0.26) for Identifying, − 0.14 (− 0.36 to 0.12) for Referral to GP, 0.23 (− 0.13 to 0.43) for Referral to 113, 1.17 (0.83 to 1.59) for Knowledge, and 0.74 (0.43 to 1.09) for Confidence.

**Table 2:** Comparison between pre-and post-training results per item

Item	Pre-training (T0)	Post-training (T1)	*F*
	Mean(SD)	Mean(SD)	
1. Identifying	1.23(1.86)	1.17(1.84)	0.36
2. Referral to GP	0.88 (1.65)	0.67(1.38)	3.89
3. Referral to 113	0.27(1.29)	0.68(2.23)	1.27
4. Knowledge	11.49(2.69)	14.30(2.11)	187.83***
5. Confidence	9.54 (2.25)	11.08(1.93)	84.44***

***p < .001

Mean scores on pre- and post-training items per employment sector are presented in Table 3. The repeated measures ANOVA with employment sector as a between subjects variable showed no interaction effect between employment sector and item scores pre- and post-training (F_identifying_(3, 150) = 0.39, *p* > .05; F_referralGP_(3, 150) = 0.75, *p* > .05; F_referral113_(3, 150) = 1.11, *p* > .05; F_knowledge_(3, 150) = 1.02, *p* > .05; F_confidence_(3, 150) = 1.01, *p* > .05).

**Table 3:** Pre- and post-training mean scores per employment sector

	Sector							
	Educa tion		Health care		Socio- economic		Other	
Item	T0 Mean (SD)	T1 Mean (SD)	T0 Mean (SD)	T1 Mean (SD)	T0 Mean (SD)	T1 Mean (SD)	T0 Mean (SD)	T1 Mean (SD)
1. Identifying	0.84 (1.11)	0.91 (1.21)	2.10 (2.46)	1.97 (2.30)	0.38 (0.62)	0.45 (1.35)	0.58 (0.77)	0.21 (0.42)
2. Referral GP	1.30 (2.40)	0.95 (1.40)	0.78 (1.13)	0.79 (1.68)	0.62 (1.43)	0.21 (0.68)	0.68 (1.34)	0.32 (0.75)
3. Referral 113	0.07 (0.26)	0.72 (1.05)	0.35 (1.48)	0.92 (3.27)	0.07 (0.37)	0.38 (1.05)	0.74 (2.42)	0.21 (0.71)
4. Knowledge	11.65 (2.43)	14.44 (1.88)	12.19 (2.32)	14.87 (1.72)	10.00 (2.79)	13.41 (2.24)	11.11 (3.38)	13.42 (2.85)
5. Confidence	9.30 (2.36)	11.14 (1.81)	10.33 (1.95)	11.56 (1.93)	8.59 (2.03)	10.17 (1.85)	8.89 (2.51)	10.74 (1.91)

To examine whether the employment sectors started off the same at baseline, post hoc tests using the Bonferroni correction [46] revealed that participants working in the healthcare sector reported a significantly higher number of people with suicidal thoughts they had identified pre- and post-training, compared to the other employment sectors (*p* < .001), and rated themselves as having more baseline knowledge and confidence compared to participants working in the socioeconomic sector (*p* = .001). Therefore, we can conclude that while participants working in the mental healthcare sector often rate themselves higher, the effect of a gatekeeper training program does not depend on the sector that participants work in and elicits a statistically significant improvement in participants’ knowledge and in their confidence in abilities to address suicide.

## Discussion

This study was conducted to evaluate the effect of a suicide prevention gatekeeper training in the Netherlands among education professionals (student career counselors, teachers, student psychologists, school social workers), designated healthcare professionals (mental healthcare professionals, GPs, social community teams, first aid workers), employees in the socioeconomic sector (bailiffs, vocational experts, bank employees, insurance doctors, debt counselors and administrators) and other professionals (security, justice, transport, church workers, imams).

The results confirm that the training was effective in all employment sectors in improving participants’ knowledge on suicide and addressing suicidality, and in their self-confidence to conduct a dialogue on suicide and suicidal thoughts. Six weeks after the gatekeeper training, all participants benefitted significantly from the training on these topics. These outcomes are consistent with other studies on gatekeeper training effects, showing primarily increased knowledge and self-confidence [16, 24–32].

With regard to our second research aim - the results from the different sectors of employment - we hypothesized that healthcare staff might be more equipped with knowledge and confidence before attending the training and consequently might have fewer benefits from the training. Indeed, participants working in the healthcare sector rate themselves as having more knowledge and confidence at baseline, compared to participants working in the socioeconomic sector. Also they score higher on identifying suicidality than participants from other sectors at baseline. Surprisingly, at follow-up all participants in all sectors self-report comparable positive results on knowledge and confidence, regardless whether they were educated in healthcare or not. In other words, professionals from various backgrounds all benefited similarly from the gatekeeper training.

Unexpectedly, no effect was found on the number of referrals to either the general practitioner or the lifeline of 113. This outcome is rather disappointing as the gatekeeper training was designed with the goal to address people with suicidal ideation and refer them to appropriate help resources. By training community gatekeepers we expected more suicidal people to be guided towards a help provider in their close vicinity, preferably their general practitioner. In addition, we estimated that 113 would become better known as a source for help by offering the training program, resulting in an increase in referrals to its services.

One way to interpret the significantly positive effect on knowledge and confidence on the one hand and the lack of effect on identifying and referral on the other, is the design of the questionnaire. It combines heterogeneous items, so the outcomes are likely to vary. It can be assumed that participants of the gatekeeper training were continuously exposed to knowledge about suicide and were encouraged to deal with the topic. Taking this into account, it is not surprising that knowledge and confidence items achieve a proximal effect, which we see in our results. On the other hand, questions about identification and referral relate to more distal effects and therefore are less likely to be found in the short period of time (about 6 weeks) between baseline and follow-up.

Another reason that no effect in referral behaviour was found can be due to the greatly diminished group of respondents at follow-up compared to baseline. The group that completed both questionnaires (*N* = 174) did not differ in baseline results from the group that completed only the baseline questionnaire (*N* = 328). In addition, all responders and nonresponders were evenly divided between included sectors: education sector (28.2% vs 24.4%), healthcare sector (40.2 vs 40.9), socioeconomic sector (18.4 vs 22.3) and other employment sectors (13.2 vs 12.5). This means that there was no differential non-response and we can rule out any disproportionate difference between responses per sectors.

Yet, the response rate of 35% at the digital follow-up survey (t1) is a manifest limitation of this study. The low percentage might be ascribed to the different ways we presented the T0 and T1 forms. T0 was handed out at the entrance to the training facilities, where all participants were asked to fill in the questionnaire right at the start of the training program. The T1 form was sent to the trainees by email. Even when sent a digital reminder to fill in the follow-up, the response numbers remained low.

A contextual explanation for the lack of effect found in referrals, could be that the gatekeepers did not have proper access to 113, a GP nearby or other help resources. It is plausible that good access to (formal or informal) help will raise the rate of referrals by gatekeepers [29]. Another possibility is that gatekeepers still felt uncomfortable about speaking extensively about suicidality, so their self-reported confidence did not lead to actual behavioural change.

At the same time, most professionals do not often meet a suicidal client; increased post-training effects on this item perhaps were less likely to occur as the time between the training and the second questionnaire was relatively short. This can also explain why no effect in identifying suicidal people was found.

The low response rate may also have influenced outcomes regarding knowledge and confidence. Although comparable international studies found similarly low response-rates in follow-up surveys [47, 48], we opted to apply Cohen’s d to measure the strength of our significantly positive findings. The effect sizes we found, subsequently demonstrate that the power of the effects on enhanced knowledge and confidence are strong: 1.17 (0.83 to 1.59) for Knowledge, and 0.74 (0.43 to 1.09) for Confidence. Another factor that may had influence on the positive outcomes on knowledge and confidence, is the method of gathering data. This was conducted with a self-report questionnaire and possibly led to socially desirable answers. Still, the choice for self-reporting is a common practice in research on trainings and appropriate in a explorative observational study.

However, the absence of a control group, the second limitation of this study, might also have caused a responders’ bias. And although our research design (non-comparative observational analysis) is not uncommon for measuring effect of an intervention in time [49], the internal validity of our effects is relatively low. Most participants voluntarily participated in our training sessions, which possibly caused a selection bias of “natural helpers” [29, 50]. Nevertheless, the effect of the gatekeeper training convincingly improves the knowledge and self-confidence of all attending professionals, accordingly we conclude the external validity of the study remains high.

When looking at the effectiveness and review studies, randomized controlled trials are exceptional [21, 23, 29, 35, 51, 52] in the research on the effect of gatekeeper trainings. Therefore, a comprehensive follow-up study according to the golden standard of randomization would be appropriate.

The few sociodemographic information that was gathered with the questionnaire holds another risk of bias and must therefore be mentioned as a limitation. At this point it is uncertain whether, for example, age, race, or gender affected the outcomes in one way or another. Notwithstanding that all sectors show the same positive outcomes on knowledge and confidence, there is the possibility that the sociodemographic distribution of the responders group was not equal to the nonresponders group.

Finally, our study is not an experimental design, therefore we have to take into account that some external factors may have influenced our results and biased our positive outcomes. In our opinion though, the short time span between baseline and follow-up makes it highly unlikely that such a determining event occurred, so the validity of our statistically positive outcomes remains.

## Conclusions

Given the limitations, our findings show that the gatekeeper training is effective in improving knowledge and skills-confidence for a wide range of professions and thus positively supports gatekeepers’ willingness to address suicidality. Moreover, dissemination of the training program could contribute to reducing the taboo on addressing suicide and suicidal thoughts in the immediate vicinity of trainees and even encourage prevention policy within institutions where staff is trained [24, 53]. Based on these considerations and the results of our study, we conclude that increasing the number of gatekeeper training programs in all sectors is recommended.

## Additional file

Additional file 1: Questionnaire Gatekeeper training. Data identifies people at risk of suicide, referral to GP, referral to 113 Suicide Prevention, knowledge and confidence. Personal data of participants and ordinal data on a 5 point scale. (DOCX 15 kb)

## Abbreviations

CBS/StatLine: Centraal Bureau voor de Statistiek, Statistics Netherlands
GLM: General linear model
GP: General practitioner
IGZ: Inspectie Gezondheidszorg en Jeugd, Dutch Health Care Inspectorate
QPR: Question, persuade, refer
