# Supplementary material for: Suicide prevention gatekeeper training in the Netherlands improves gatekeepers’ knowledge of suicide prevention and their confidence to discuss suicidality, an observational study
Source: BMC Public Health. 2018 May 18;18:637. doi: 10.1186/s12889-018-5512-8 (PMC5960185; doi:10.1186/s12889-018-5512-8)
Supplement: Supplementary file 1 — Questionnaire Gatekeeper training. Data identifies people at risk of suicide, referral to GP, referral to 113 Suicide Prevention, knowledge and confidence. Personal data of participants and ordinal data on a 5 point scale. (DOCX 15 kb) [file 12889_2018_5512_MOESM1_ESM.docx]

*Questionnaire Gatekeeper training*

| 01 | Date |  |
| --- | --- | --- |
| 02 | Name |  |
| 03 | E-mail address |  |
| 04 | Profession or organization |  |
| A1 | How often have you spoken to someone with suicidal thoughts in the past 4 weeks? (number of people) |  |
| A2 | How often have you referred someone to seek help at a general practitioner (GP) or another healthcare professional in the past 4 weeks? (number of people) |  |
| A3 | How many people have you referred to 113 Suicide Prevention? (number of people) |  |
| *How much do you know about the following subjects:* |  |  |
| B1 | Knowledge on suicide | 5 point scale:  very few – very much |
| B2 | Signals that point out suicidal behavior | 5 point scale:  very few – very much |
| B3 | How to talk to someone on his/her suicidal thoughts | 5 point scale:  very few – very much |
| B4 | How to arrange help for a suicidal person | 5 point scale:  very few – very much |
| *Indicate to which extent the following conditions apply to you:* |  |  |
| C1 | I have confidence in my abilities to assess the suicidal risk for clients | 5 point scale:  not at all – a lot |
| C2 | I have confidence in my abilities to handle suicidal clients correctly | 5 point scale:  not at all – a lot |
| C3 | I hesitate to ask a client whether he / she is suicidal | 5 point scale:  not at all – a lot |
